# Analysis of the characteristics and illness comprehension bias among Chinese patients with psycho-cardiovascular disease: a multi-centre cross-sectional survey

**DOI:** 10.7189/jogh.15.04019

**Published:** 2025-01-31

**Authors:** Zhuofei Shi, Kun Xia, Jianchao Li, Jianqi Lu, Hongping Lu, Yanli Li, Jifeng Zhang, Qilan Chen, Jing Liu, Rongjing Ding

**Affiliations:** 1 Department of Cardiology, Peking Union Medical College Hospital, Chinese Academy of Medical Sciences, Beijing, China; 2 Beijing Chaoyang Hospital Affiliated to Capital Medical University Heart Centre, Beijing, China; 3 Beijing Advanced Innovation Centre for Biomedical Engineering, Beihang University, Beijing, China; 4 Department of Cardiology, The First Affiliated Hospital of Guangxi University of Chinese Medicine, Nanning, China; 5 Department of Cardiology, Nanning Red Cross Hospital, Nanning, China; 6 Department of Cardiology, Jiamusi Central Hospital, Jiamusi, China; 7 Department of Cardiology, Zhejiang Provincial Litongde Hospital, Hangzhou, China; 8 Department of Cardiology, Hangzhou Hospital of Traditional Chinese Medicine, Hangzhou, China; 9 Department of Cardiology, The Fourth Affiliated Hospital of China Medical University, Shenyang, China

## Abstract

**Background:**

Psychological distress, such as depression and anxiety, impacts cardiovascular disease (CVD) prognosis and management. Illness comprehension is essential for effective treatment, but biases can lead to suboptimal outcomes. We explored psycho-cardiovascular disease (PCD) patient characteristics, with a specific focus on comprehension biases and treatment choices from patients’ perspectives in China, to improve management strategies.

**Methods:**

We enrolled 864 PCD patients in Chinese hospitals across 11 provinces. Tools included the seven-item General Anxiety Disorder scale, the nine-item Patient Health Questionnaire, and a self-designed PCD illness comprehensibility survey. We used χ^2^ test, univariate, and multivariate logistic regression to examine patient characteristics.

**Results:**

Of 834 enrolled PCD patients, over 90% experienced mild to moderate anxiety and depression, yet less than 10% received treatment. 52.90% of patients had high illness comprehension. Among the high comprehension group, there were fewer labourers (19.30% *vs.* 26.40%; *P* < 0.05), fewer older individuals (39.20% *vs.* 46.90%; *P* < 0.05), and those with lower household income (15.60% *vs.* 30.50%; *P* < 0.05). A greater proportion of those in the high comprehension group lacked insurance (17.50% *vs.* 10.00%; *P* < 0.05), and they were more highly educated (42.90% *vs.* 32.10% with a college education). Additionally, more patients in the high comprehension group frequently received psychological consultation (24.00% *vs.* 5.10%; *P* < 0.05) and therapy (7.70% *vs.* 2.30%; *P* < 0.05). These patient groups preferred tertiary hospitals (71.66% *vs.* 63.33%; *P* < 0.05) and psycho-cardiovascular clinics (40.14% *vs.* 25.90%; *P* < 0.05). In comparison, low comprehension patients prioritised cost (32.65% *vs.* 46.41%; *P* < 0.05) and favoured a transition to community hospitals (16.55% *vs.* 25.38%; *P* < 0.05).

**Conclusions:**

More than 90% of PCD patients in Chinese CVD departments experience mild to moderate anxiety and depression with low treatment rates. Different illness comprehension levels are associated with variations in treatment willingness, considerations, health care preferences, medication choices, and illness knowledge acquisition methods.

Cardiovascular diseases (CVDs) rank among the leading causes of death worldwide, with an age-standardised mortality rate of 239.90 per 100 000 people globally and 245.39 per 100 000 in China [1,2]. Growing evidence indicates a complex interplay between CVDs and mental health issues, such as depression and anxiety [3]. A study published in the Journal of the American Medical Association, which involved 22 cohorts and 563 255 participants, revealed that depression is associated with a higher risk of CVD (hazard ratio (HR) = 1.16; 95% confidence interval (CI) = 1.04–1.30), leading to increased all-cause mortality (HR = 1.43; 95% CI = 1.27–1.60) and CVD mortality (HR = 1.44; 95% CI = 1.27–1.63) [4]. Depression is significantly correlated with the incidence of coronary heart disease (CHD), leading to a 60–80% increased risk of developing CHD [5]. Anxiety is also a risk factor for CVDs [6] and is significantly associated with cardiovascular death (relative risk (RR) = 1.41; 95% CI = 1.13–1.76) and the risk of CHD (RR = 1.41; 95% CI = 1.23–1.61) [7]. Therefore, recognising and managing these psychological risk factors and identifying key influencing elements is crucial for informing targeted strategies to improve health outcomes.

As the connection between social psychological factors and CVD becomes more pronounced, research in psycho-cardiovascular disease (PCD) is gradually gaining public attention [8]. Some studies have investigated the factors associated with depression and anxiety in patients with cardiovascular disease, most of which have approached the characteristics of PCD from a physician’s perspective, with a limited understanding of how PCD patients perceive the impact of depression and anxiety on CVD, as well as how this understanding influences their preferred treatment choices [9,10]. Prior research indicates that individuals manage health threats (such as heart attacks) through a dynamic self-regulatory process, which includes illness perception. Illness perception can guide subsequent actions and strategies for disease management [11]. Negative illness perception was linked with psychological distress (such as depression, anxiety, and acute stress disorder), fatigue, poor adherence to health behaviours (such as exercise and participation in cardiac rehabilitation), reduced quality of life, and longer time needed to return to work in CVD patients [12]. A positive perception of illness has been shown to improve these outcomes [13–16], as well as enhance satisfaction with diagnosis and treatment [17].

There is a notable lack of research on illness perception among patients in both Western countries and China. Due to cultural differences, findings from Western studies may not be directly applicable to patients in China. Therefore, to better improve the psychological health and physical condition of Chinese PCD patients, it is essential to gain a deeper understanding of how these patients perceive PCD in China and investigate characteristics among them.

## METHODS

### Aims

Our primary aim was to investigate the illness comprehension biases of PCD patients, focusing on their demographic characteristics and differences in treatment choices based on these biases. The secondary aim was to assess the levels of anxiety and depression in PCD patients and to explore the influencing factors after adjusting for demographic and clinical variables.

### Research procedure

This is a multi-centre, cross-sectional, self-administered survey study conducted in Chinese tertiary and secondary hospitals from 26 June to 31 July 2023. Regarding sample size, since the level of understanding of PCD among patients is unknown, we assumed that the expected proportion of those with a high level of understanding is 50%, with a permissible error of 0.05. To achieve a desired power of 0.80 and an α of 0.05, we estimated that we would need 784 participants to detect this characteristic proportion. Considering potential participant non-response, with a non-response rate of 6% observed in the pilot study, the final estimated required sample size was approximately 835.

We used convenience sampling to recruit PCD patients in cardiovascular outpatient departments across 11 provinces. We used the following criteria to determine eligibility for inclusion: a diagnosis of CHD confirmed through medical records (either outpatient or hospitalisation) or by diagnostic methods such as electrocardiogram, exercise tests, echocardiography, or coronary angiography, and exhibiting symptoms of anxiety or depression, as assessed by the seven-item General Anxiety Disorder (GAD-7) scale and nine-item Patient Health Questionnaire (PHQ-9), with a total score of ≥5 on both scales. Exclusion criteria were: 1) refusal to participate in the survey; 2) individuals affected by mental or language barriers that hinder data collection; 3) pregnant or breastfeeding women; and 4) any other factors that may compromise the authenticity of the survey results.

Trained cardiovascular specialists screened potential participants and provided a comprehensive introduction to the study’s objectives, methods, and potential benefits. After obtaining informed consent from the patients, they were guided in completing the online questionnaire, with all questions addressed patiently to ensure a thorough understanding of the research content. In the entire recruitment and consent process, we strictly adhered to ethical review requirements to safeguard the study’s legality and participants’ rights. We collected a total of 864 questionnaires through an electronic format.

Research Ethics Service of Peking University People’s Hospital approved the study protocol, and all participants provided informed consent, which explicitly stated that personal information and health data would be used solely for this study and that strict confidentiality measures would be implemented.

### Quality control

A total of 50 PCD patients participated in the pilot testing of the questionnaire. Based on their feedback, we modified the questionnaire items and wording, resulting in the final version. We designed the survey items to be clear and straightforward, focusing solely on patients’ illness comprehension of PCD without addressing sensitive issues. All questionnaire items were set as mandatory to minimise non-response rates and enhance completeness. In selecting participants, we strictly adhered to the inclusion and exclusion criteria to mitigate the risk of selection bias. Additionally, we implemented measures to reduce the risk of reporting bias, including assurances of participant privacy and data confidentiality. We adopted anonymisation procedures to prevent any linkage of responses to individual participants. We stored data in an encrypted format with access controls to limit access to authorised personnel only. We also utilised secure data transmission protocols to protect the information during transfer. Furthermore, all researchers involved in data collection and processing received professional training and supervision to ensure compliance with ethical standards. We assured the participants that the questionnaire would be answered anonymously, their information would remain confidential, and the results would be used solely for research purposes. Furthermore, completing the questionnaire would not impact their treatment process or any other comprehensive evaluations.

### Survey tool

#### Chinese PCD patient comprehension questionnaire

Experts in the field of PCD designed the questionnaire in collaboration with the researchers, to align with the research objectives. The questionnaire comprised several sections. First, a screening section for patients with CHD and comorbid anxiety/depression. We utilised the GAD-7 scale in this study to evaluate anxiety symptoms. Scores ranged from 0–21, with thresholds for mild (score 5), moderate (score 10), and severe (score 15) anxiety [18]. We also utilised the PHQ-9 in this study to evaluate symptoms of depression. Scores ranged from 0–27, with thresholds for mild (score 5), moderate (score 10), moderate-to-severe (score 15), and severe (score 20) depression [19]. Further, the questionnaire contained a section related to general demographic data (name, gender, age group, height, weight, current residence, marital status, occupation, monthly income, and medical insurance), as well as general medical history and diagnosis data, including cardiovascular-related medical history, disease duration, psychological consultation and therapy, and current efficacy evaluation. Finally, the questionnaire contained a section on the illness comprehensibility of PCD patients, covering the understanding of PCD, the impact of psychological factors on CVD, and surveys on willingness related to integrated cardio-psychiatric treatment.

Before the actual data collection period, we conducted a pilot test of the questionnaire with 50 participants. The pilot test results indicated that the questionnaire demonstrated good reliability and validity, with a Cronbach’s α coefficient of 0.81, a content validity index of 0.85, and a Bartlett’s test *P*-value <0.05. These findings suggested that the questionnaire suits related research and practical applications.

#### Statistical analysis

Continuous variables were expressed as means and standard deviations and, if normally distributed, medians and interquartile ranges. Categorical variables were expressed as absolute numbers and percentages. We did not employ imputation methods. Instead, we utilised listwise deletion, as the missing data comprised less than 5% and were determined to be random, not systematically affecting the outcomes. Based on the question ‘comprehension degree of PCD,’ we classified those with a high level of understanding, defined as ‘very familiar’ or ‘familiar with but never in touch with,’ as the high comprehension group. Patients ‘not familiar with PCD’ were in the low comprehension group. When evaluating group variances based on comprehension level, we analysed categorical variables using the χ^2^ test, while we assessed the continuous variables using the Wilcoxon rank sum test. We compared the anxiety and depression levels among PCD patients with different characteristics using the Wilcoxon rank-sum test for two-group comparisons and the Kruskal-Wallis H-test for multiple comparisons. To identify the factors influencing depression or anxiety in patients with PCD, we conducted univariate ordered multinomial logistic regression analysis and multivariate ordered multinomial logistic regression analysis. We conducted all tests bilaterally, with a statistically significant threshold of *P* < 0.05. For all calculations, we used *R*, version 4.3.1 (R Core Team, Vienna, Austria).

## RESULTS

### General information and characteristics of PCD patients

**Figure 1:**
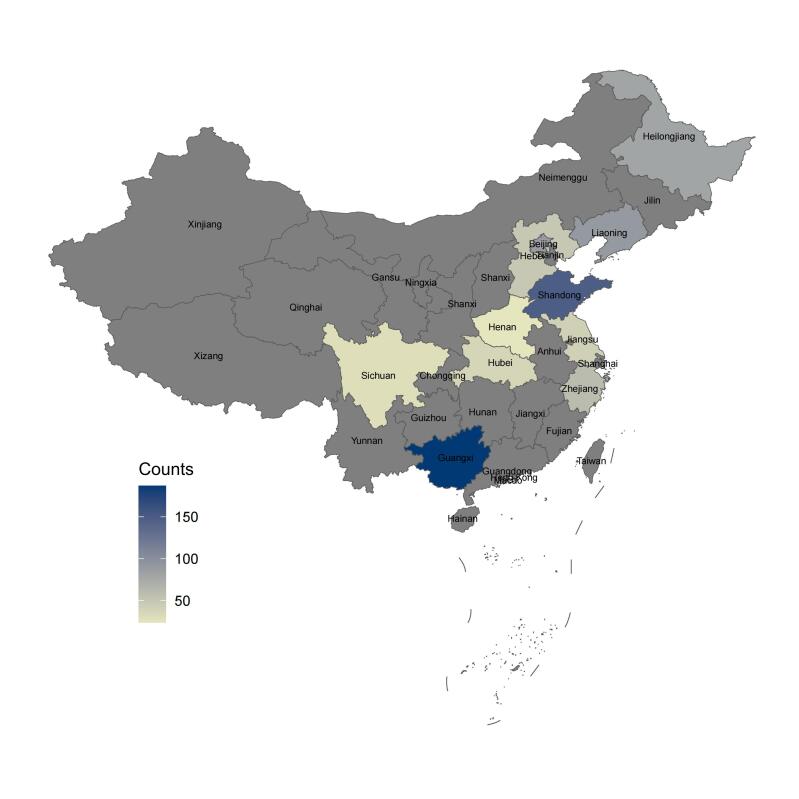
Heat map showing the regional distribution of data sources from multi-centre surveys in China.

We collected 864 samples from 11 provinces in China and excluded 30 samples with invalid responses according to the set criteria. The remaining 834 pieces of data were valid (**Figure 1**). The effective recovery rate of the questionnaire was 96.50%.

We characterised research participants based on general demographic information, general medical history, and diagnosis and treatment details (Table S1 in the **Online Supplementary Document**).

Most participants were aged >40 years (94.00%). Half were male (51.40%), and 57.40% were from North China. The majority did not receive psychological consultation (84.90%) or psychotherapy (94.80%). 58.50% of participants were satisfied with their current treatment, and 37.20% had a general evaluation of their current treatment. 52.90% of participants had a high level of illness comprehension. GAD-7 scores in PCD patients ranged from 5–16, averaging 8.29, with 73.00% experiencing mild anxiety and 26.10% experiencing moderate anxiety. PHQ-9 scores ranged from 5–20, averaging 9.81, with 48.10% experiencing mild depression and 47.20% experiencing moderate depression.

### Analysis of influencing factors for anxiety and depression in patients with PCD

Multivariate analysis confirmed that the illness duration of one to three years (odds ratio (OR) = 0.49; 95% CI = 0.27–0.88, *P* = 0.016) and four to 10 years (OR = 0.51; 95% CI = 0.28–0.94, *P* = 0.032), psychological consultation (OR = 0.40; 95% CI = 0.17–0.82, *P* = 0.018), and southern region (OR = 0.08; 95% CI = 0.04–0.13, *P* < 0.001) were related to lower anxiety (Table S2 in the **Online Supplementary Document**).

Multivariate analysis confirmed that the illness duration >10 years (OR = 0.29; 95% CI = 0.15–0.58, *P* < 0.001) and southern region (OR = 0.38; 95% CI = 0.27–0.52, *P* < 0.001) were related to lower depression levels. Additionally, more cardiovascular-related medical histories (OR = 1.62; 95% CI = 1.41–1.86, *P* < 0.001) were associated with higher depression levels (Table S3 in the **Online Supplementary Document**).

### The effect of comprehension degree on patients with PCD

#### Basic information between high and low comprehension groups among PCD patients

The analysis of inter-group differences revealed significant variations in occupation, age, medical insurance, education level, and monthly household income (*P* < 0.05). The high comprehension group had a lower proportion of labourers (19.30% *vs.* 26.40%; *P* < 0.05), fewer patients aged at least 61 years (39.20% *vs.* 46.90%; *P* < 0.05), fewer patients with a monthly household income below CNY 4000 (15.60% *vs.* 30.50%; *P* < 0.05), and had a higher proportion of patients without medical insurance (17.50% *vs.* 10.00%; *P* < 0.05) and with college education (42.90% *vs.* 32.10%; *P* < 0.05), or higher education level (2.50% *vs.* 1.00%; *P* < 0.05) (**Table 1**).

**Table 1:** The analysis of high or low levels of comprehension of basic information*

Characteristics	High comprehension (n = 441)	Low comprehension (n = 390)	*P*-value
Gender			0.901
*Male*	228 (51.70)	199 (51.00)	
*Female*	213 (48.30)	191 (49.00)	
BMI in kg/m^2^			0.574
*<24*	167 (37.90)	140 (35.90)	
*24–28*	209 (47.40)	185 (47.40)	
*≥28*	59 (13.40)	62 (15.90)	
*Missing*	6 (1.40)	3 (0.80)	
Marital status			0.304
*Married*	384 (87.10)	328 (84.10)	
*Single*	55 (12.50)	59 (15.10)	
*Missing*	2 (0.50)	3 (0.80)	
Medical insurance			0.002
*Yes*	347 (78.70)	338 (86.70)	
*No*	77 (17.50)	39 (10.00)	
*Missing*	17 (3.90)	13 (3.30)	
Age in years			0.033
*30–40*	31 (7.00)	16 (4.10)	
*41–60*	235 (53.30)	190 (48.70)	
*≥61*	173 (39.20)	183 (46.90)	
*Missing*	2 (0.50)	1 (0.30)	
Occupation			<0.001
*Laborer*	85 (19.30)	103 (26.40)	
*National business unit personnel*	174 (39.50)	90 (23.10)	
*Unemployed*	181 (41.00)	195 (50.00)	
*Missing*	1 (0.20)	2 (0.50)	
Education level			<0.001
*Primary school degree or below*	31 (7.00)	50 (12.80)	
*Secondary school degree*	208 (47.20)	206 (52.80)	
*University degree*	189 (42.90)	125 (32.10)	
*Master’s degree or above*	11 (2.50)	4 (1.00)	
*Missing*	2 (0.50)	5 (1.30)	
Monthly household income in CNY			<0.001
*<4000*	69 (15.60)	119 (30.50)	
*4000–7999*	252 (57.10)	181 (46.40)	
*8000–12 000*	95 (21.50)	65 (16.70)	
*>12 000*	23 (5.20)	22 (5.60)	
*Missing*	2 (0.50)	3 (0.80)	
Region			0.609
*North*	249 (56.50)	228 (58.50)	
*South*	192 (43.50)	162 (41.50)	

BMI – body mass index

*Presented as n (%) unless specified otherwise.

#### Mental severity and treatment status between high and low comprehension group in patients with PCD

Regarding the mental severity and treatment status in patients with high or low comprehension, the high comprehension group had a higher proportion of moderate depression (52.80% *vs.* 41.30%; *P* < 0.05), while there was no difference regarding anxiety. A higher proportion of patients sought psychological consultation (24.00% *vs.* 5.10%; *P* < 0.05) and psychotherapy (7.70% *vs.* 2.30%; *P* < 0.05). More patients believed that psychological factors had a noticeable (26.50% *vs.* 24.10%; *P* < 0.05) or certain (60.80% *vs.* 49.70%; *P* < 0.05) influence on CVD, with a short duration of illness (<1 year: 9.50% *vs.* 8.30%; 1–3 years: 56.50% *vs.* 46.90%; *P* < 0.05), more cardiovascular-related medical histories (3.33 *vs.* 2.74; *P* < 0.05). A higher proportion of patients were very satisfied (20.20% *vs.* 15.60%; *P* < 0.05), and a lower proportion of patients were dissatisfied (2.50% *vs.* 5.90%; *P* < 0.05) (**Table 2**).

**Table 2:** The analysis of different comprehension degrees on mental severity and treatment status of PCD patients*

Characteristics	High comprehension (n = 441)	Low comprehension (n = 390)	*P*-value
Three-question symptom†			0.797
*x̄ (SD)*	2.16 (0.84)	2.16 (0.82)	
*MD (IQR)*	2.00 (0.00–3.00)	2.00 (0.00–3.00)	
Cardiovascular-related medical history			<0.001
*x̄ (SD)*	3.33 (1.37)	2.74 (1.11)	
*MD (IQR)*	3.00 (1.00–8.00)	3.00 (1.00–6.00)	
Duration of illness in years			0.015
*<1*	42 (9.50)	32 (8.20)	
*1–3*	249 (56.50)	183 (46.90)	
*4–10*	109 (24.70)	131 (33.60)	
*>10*	41 (9.30)	44 (11.30)	
Degree of anxiety			0.403
*Mild*	325 (73.70)	281 (72.10)	
*Moderate*	114 (25.90)	104 (26.70)	
*Severe*	2 (0.50)	5 (1.30)	
Degree of depression			<0.001
*Mild*	183 (41.50)	215 (55.10)	
*Moderate*	233 (52.80)	161 (41.30)	
*Moderate to severe*	25 (5.70)	13 (3.30)	
*Severe*	0 (0.00)	1 (0.30)	
Psychological consultation			<0.001
*Yes*	106 (24.00)	20 (5.10)	
*No*	335 (76.00)	370 (94.90)	
Psychological therapy			<0.001
*Yes*	34 (7.70)	9 (2.30)	
*No*	407 (92.30)	381 (97.70)	
Current efficacy evaluation			0.021
*Very satisfied*	89 (20.20)	61 (15.60)	
*Satisfied*	176 (39.90)	160 (41.00)	
*General*	165 (37.40)	146 (37.40)	
*Not satisfied*	8 (1.80)	22 (5.60)	
*Very dissatisfied*	3 (0.70)	1 (0.30)	
Impact of psychological factors on CVD			<0.001
*Noticeable*	117 (26.50)	94 (24.10)	
*Certain*	268 (60.80)	194 (49.70)	
*Minor*	49 (11.10)	93 (23.80)	
*No impact*	4 (0.90)	6 (1.50)	
*Missing*	3 (0.70)	3 (0.80)	

CVD – cardiovascular disease, IQR – interquartile range, MD – median, SD – standard deviation, x̄ – mean

*Presented as n (%) unless specified otherwise.

†The ‘three-question symptom’ is a method used by Chinese cardiologists to initially screen for possible CVD and mental health problems during or before diagnosis. The ‘three questions’ are as follows: 1) Is there poor sleep that has significantly affected the mental state during the day or requires medication? 2) Is there a feeling of restlessness and loss of interest in previously interesting things? 3) Is there any obvious physical discomfort, but multiple examinations have not found any reason to explain organic cardiovascular disease? If two out of three questions are answered with ‘yes,’ the likelihood of mental disorders is about 80% [20].

#### The effect of high or low comprehension degree on PCD patients’ treatment choice

Results showed that the majority of patients (98.32%) were willing to undergo psycho-cardiovascular therapy. When considering treatment choice, hospital level (63.67%) and doctor’s credibility (58.15%) were the primary concerns. Patients with high comprehension preferred emphasis on hospital level (68.03% *vs.* 58.72%; *P* < 0.05) and the availability of psycho-cardiovascular clinics (40.14% *vs.* 25.90%; *P* < 0.05), while patients with low comprehension preferred emphasis on treatment cost (32.65% *vs.* 46.41%; *P* < 0.05). More patients with high comprehension tended to choose tertiary hospitals exclusively (71.66% *vs.* 63.33%; *P* < 0.05), yet patients with low comprehension favoured early-stage treatment in a tertiary hospital, followed by a community hospital (16.55% *vs.* 25.38%; *P* < 0.05). Most patients preferred treatment by cardiologists (72.18%) or psycho-cardiologists (70.86%). A preference for integrated Chinese and Western medicine was observed (69.90%), with high comprehension patients favouring sole Chinese medicine treatment (48.07% *vs.* 33.59%; *P* < 0.05) (**Table 3**).

**Table 3:** The effect of PCD comprehension on treatment choice of patients with PCD*

Questions	Overall	High comprehension (n = 441)	Low comprehension (n = 390)	*P*-value
Willingness to receive integrated psycho-cardiovascular treatment				0.106
*Willing*	820 (98.32)	431 (97.73)	386 (98.97)	
*Not willing*	11 (1.32)	9 (2.04)	2 (0.51)	
Degree of intention for integrated psycho-cardiovascular treatment				0.001
*CVD but no other symptoms*	253 (30.34)	160 (36.28)	92 (23.59)	
*CVD accompanied by symptoms such as tension, anxiety, sleep disorders, loss of appetite, and decreased energy*	766 (91.85)	392 (88.89)	371 (95.13)	
Main factors considered for accepting integrated psycho-cardiovascular treatment				<0.001
*Hospital level*	531 (63.67)	300 (68.03)	229 (58.72)	
*Whether the hospital has an integrated psycho-cardiovascular clinic*	279 (33.45)	177 (40.14)	101 (25.90)	
*Proximity of the hospital*	229 (27.46)	117 (26.53)	111 (28.46)	
*Follow-up frequency*	74 (8.87)	45 (10.20)	29 (7.44)	
*Cost*	326 (39.09)	144 (32.65)	181 (46.41)	
*Doctor’s credibility*	485 (58.15)	252 (57.14)	231 (59.23)	
*Treatment plan*	341 (40.89)	175 (39.68)	164 (42.05)	
Type of hospital for accepting integrated psycho-cardiovascular treatment				0.010
*Tertiary*	566 (67.87)	316 (71.66)	247 (63.33)	
*Secondary*	307 (36.81)	173 (39.23)	134 (34.36)	
*Community*	51 (6.12)	30 (6.80)	21 (5.38)	
*Early stage in a tertiary hospital, followed by a community hospital*	172 (20.62)	73 (16.55)	99 (25.38)	
Type of doctor for accepting integrated psycho-cardiovascular treatment				0.098
*Cardiology department*	602 (72.18)	303 (68.71)	298 (76.41)	
*Integrated psycho-cardiovascular doctor (a cardiologist who understands psychology)*	591 (70.86)	333 (75.51)	257 (65.90)	
*Psychiatrist*	13 (1.56)	8 (1.81)	5 (1.28)	
*Psychologist*	150 (17.99)	91 (20.63)	59 (15.13)	
*General practitioner*	119 (14.27)	68 (15.42)	51 (13.08)	
Types of recent physical exercise				0.090
*No exercise*	128 (15.35)	64 (14.51)	63 (16.15)	
*Walking, doing exercises*	557 (66.79)	299 (67.80)	256 (65.64)	
*Jogging*	140 (16.79)	69 (15.65)	71 (18.21)	
*Traditional practices such as Tai Chi*	172 (20.62)	109 (24.72)	63 (16.15)	
*Badminton, basketball, tennis*	96 (11.51)	56 (12.70)	40 (10.26)	
*Other*	18 (2.16)	12 (2.72)	6 (1.54)	
Medication preferences for treating integrated PCD				0.003
*Traditional Chinese medicine*	343 (41.13)	212 (48.07)	131 (33.59)	
*Western medicine*	77 (9.23)	35 (7.94)	42 (10.77)	
*Combination of Chinese and Western medicine*	583 (69.90)	300 (68.03)	280 (71.79)	
Preferred ways to acquire knowledge on preventing and treating integrated PCD				0.004
*Online videos*	382 (45.80)	246 (55.78)	136 (34.87)	
*WeChat public accounts*	276 (33.09)	153 (34.69)	123 (31.54)	
*Television and radio broadcasts*	182 (21.82)	89 (20.18)	93 (23.85)	
*Newspapers, magazines, books*	214 (25.66)	122 (27.66)	91 (23.33)	
*Patient education manuals*	312 (37.41)	178(40.36%)	132 (33.85)	
*Patient clubs*	74 (8.87)	33 (7.48)	40 (10.26)	
*Doctor education*	619 (74.22)	329 (74.60)	288 (73.85)	
*Other*	4 (0.48)	3 (0.68)	1 (0.26)	

CVD – cardiovascular disease, PCD – psycho-cardiovascular disease

*Presented as n (%) unless specified otherwise.

The education choices of patients regarding PCD prevention and treatment included doctor education as the most preferred option (74.22%), followed by online videos (45.80%), patient education manuals (37.41%), and WeChat public accounts (33.09%). A higher proportion of patients with high comprehension preferred online videos (55.70% vs 34.80%; *P* < 0.05) (**Table 3**).

## DISCUSSION

In this study, we provided current evidence on how Chinese patients with PCD perceive the impact of depression and anxiety on CVD and their preferences for treatment. We found that more than 90% of PCD patients in Chinese CVD departments experienced mild to moderate anxiety/depression, and the treatment rates were low. Among the high comprehension group, there were fewer labourers, a smaller proportion of older individuals, and a lower percentage of those with household incomes below the median. Additionally, a greater share of this group lacked insurance and included more highly educated individuals. They also exhibited less mild depression and a higher rate of patients receiving psychological consultation and therapy. Patients in the high comprehension group reported higher satisfaction levels, with more being very satisfied and fewer expressing dissatisfaction with the current efficacy evaluation. An overwhelming majority were willing to pursue psycho-cardiovascular therapy, showing a preference for either cardiologists or psycho-cardiovascular specialists. In terms of treatment considerations, the primary factors influencing choice included hospital level and doctor credibility. The high comprehension group prioritised hospital level, particularly favouring tertiary hospitals and psycho-cardiovascular clinics, while also showing a preference for Chinese medicine. Conversely, patients in the low comprehension group prioritised treatment costs and favoured a shift from tertiary to community hospitals. Patients sought information about PCD primarily through doctor education, videos, and WeChat.

In this multi-centre survey, we found that anxiety and depression among PCD patients were predominantly mild to moderate, with a treatment rate of less than 10%. Our findings added more data to the previous studies. A previous study showed that depression and anxiety were frequently overlooked and inadequately treated, leading to frequent misdiagnosis during hospitalisation and persistence of symptoms after discharge [21]. A study in the European Journal of Preventive Cardiology, which involved 7589 patients with CHD from 24 European countries, revealed that 26.3% of participants exhibited symptoms of anxiety and 22.4% exhibited symptoms of depression. However, only 2.4% of patients were prescribed antidepressants and anxiolytics, and 2.7% and 5.0% of patients, respectively, continued to take these medications at follow-up [22]. A recent study of 364 patients with CHD in Germany showed that only 9.1% reported receiving assistance in seeking psychotherapy or pharmacotherapy (4.1%) [23]. Despite the high prevalence and poor prognosis of depression on CVD, a previous cross-sectional epidemiological study revealed that the treatment rate of PCD in China was meagre (2.4%) [24]. We strengthened the idea that the assessment of depression and anxiety symptoms should be integrated into the clinical management of cardiovascular departments. To achieve this, clinicians and staff should receive training on mental health assessments (via workshops, seminars, and continuing education), and standardised screening protocols using validated tools like PHQ-9 and GAD-7 should be established. Multidisciplinary collaboration with mental health professionals is crucial, and patient education about the impact of depression and anxiety on cardiovascular health is essential. Clear clinical guidelines and policies must be developed to institutionalise this practice, and regular monitoring and evaluation of the implementation and impact on patient outcomes should be conducted.

We found that geographical discrepancies in anxiety and depression among PCD patients existed, which may be linked to regional economic development. Studies in China indicated that economically advanced coastal areas experienced reduced cardiovascular disease burdens [25], with heightened economic development aiding in alleviating patient anxiety and depression levels [26–28], which may explain the finding in our study of lower anxiety and depression in the south of China. Love et al. findings suggested that psychological resilience plays a pivotal role in cardiovascular patients’ psychological well-being [29]. Individuals with greater resilience can better manage and adapt to their illness, thus lowering anxiety and depression levels. Patients with longer illness duration may cultivate stronger psychological resilience, enabling better anxiety and depression management [30]. We testified to the conclusion above that longer illness duration was associated with lower anxiety and depression levels.

Currently, there is still limited research on the illness perception of patients with PCD. To gain a deeper understanding of the impact of high or low illness comprehension on patients, this study conducted a grouping analysis based on the degree of comprehension of PCD. We found that non-manual labour occupations, younger age, higher education level, higher income, shorter illness duration, and more cardiovascular-related medical histories are related to higher disease comprehension, consistent with previous studies [31–35]. Our study results also indicated that people with higher comprehension of PCD tended to lack medical insurance, experience higher levels of depression, receive more psychological interventions (including psychological consultation and psychotherapy), report higher satisfaction with treatment, and have a deeper impact of psychological factors on CVD. Our study aligned with previous studies. A study involving 510 Chinese patients with CHD revealed that, after adjusting for potential confounding factors, a one-point increase in illness perception scores (ranging from zero to 10) related to illness consequences and emotional reactions was linked to a 22% or 38% higher likelihood of depressive or anxiety symptoms [36]. Beyond illness perception, social support also served as a mediator between CVD and depression [37]. Patients with poor social support tended to exhibit more depressive symptoms [38]. These factors may explain why patients with high illness comprehension experienced higher levels of depression, given their lack of social support (medical insurance) and the deeper impact of psychological factors on CVD. Patients with a higher perception of illness have greater mental health needs, leading to a higher proportion of seeking and receiving psychological interventions such as psychological consultation and psychotherapy [39]. We found that patients with high PCD comprehension tended to have greater satisfaction with the treatment, which may be attributed to earlier treatment, effective treatment, as well as patients' education about the relationship between CVD and mental disorders [40]. Studies showed that disease comprehension correlated with prevention attitudes, affecting disease management and treatment adherence [41,42]. Enhancing disease comprehension can promote health screenings, preventive behaviours, early disease prevention, effective management, and improved quality of life [42,43]. Our results reminded us that psychological education for patients was important to increase illness comprehension and improve treatment satisfaction.

In our survey, we showed that 98.32% of patients were willing to undergo psycho-cardiovascular therapy. This result indicated that more efforts were needed to improve psycho-cardiovascular clinical practice and meet the needs of patients. We found that hospital and physician accreditation were key factors for patients’ treatment decisions, with 63.67% of them choosing hospital tier and 58.15% prioritising doctor’s credibility, reflecting their focus on health care quality and professionalism. The high comprehension group placed more importance on the hospital tier. Yet, patients with low comprehension tended to choose an ‘early stage in a tertiary hospital, followed by a community hospital’ treatment, reflecting their reliance on large hospital resources and trust in the rehabilitation capacity of community hospitals. This finding underscored the need to further strengthen the development and service capabilities of community hospitals to provide patients with more convenient and efficient rehabilitation services. The low comprehension of PCD patients focused more on treatment costs, likely due to economic pressures and concerns about treatment expenses. These differences suggested that health care providers should develop flexible treatment plans and cost policies tailored to the diverse needs and priorities of patients.

We showed that patients preferred cardiologists who have skills in both cardiovascular and psychological dimensions, with 72.18% preferring internal medicine cardiologists and 70.86% preferring psycho-cardiovascular doctors. Considering the patients’ clinic choice and the majority (more than 90%) of CVD patients with mild or moderate depression/anxiety, cardiologists should increase their basic knowledge of mental health and the required skills to identify and provide symptomatic treatment for CVD patients with depression and anxiety. Existing limited research evidence indicated that the combined treatment of traditional Chinese medicine and Western medicine often yielded better therapeutic effects on patients than Western medicine alone, with fewer adverse reactions occurring [44,45]. 41.13% of patients preferred traditional Chinese medicine treatment. Recent studies have shown that traditional Chinese medicines such as Guanxindanshen dropping pill [46], Xinkeshu [47], Chaihujialonggumulitang [48], and Shuangxinfang [49] may alleviate the condition and symptoms of anxiety and depression. This finding provided a basis for medical service providers to consider patient preferences and levels of illness comprehension when formulating treatment plans.

In addition to face-to-face medical education, our results showed that patients were inclined to obtain prevention and treatment knowledge of PCD through online channels, such as online videos (45.80%) and WeChat official accounts (33.09%). This suggested that when providing health education, we should fully utilise multiple channels and forms to meet the information acquisition needs of different patients. Furthermore, given the proven effectiveness of remote cardiac rehabilitation for patients [50,51], it might be worthwhile to consider establishing a similar online platform for the prevention and treatment of PCD. This could involve utilising digital technology to guide and enhance patients’ physical and mental well-being and quality of life.

This study may be affected by recall bias because it relied on self-reported data, even though the questionnaires used were carefully validated, and participants were encouraged to report information accurately. The cross-sectional design of the study does not allow for the establishment of causal relationships between specific factors and levels of depression, anxiety, or illness comprehension. It also limits the ability to observe changes over time in PCD patients’ mental health and illness comprehension. Further research with a longitudinal design is necessary to make causal inferences and provide insights into how these factors evolve and impact treatment choices and health outcomes. Besides, potential confounding factors were not considered, which introduced the possibility of confounding. This suggested that, apart from the variables being studied, there may be other unmeasured or uncontrolled variables affecting the research outcomes, such as lifestyle and cardiovascular medication. Moreover, there is a potential selection bias inherent in the study's design. The recruitment of patients from hospitals may not represent the broader population of individuals with PCD. This could limit the generalisability of the findings to all PCD patients in China. Future studies should explore more diverse recruitment strategies to improve representativeness and ensure broader applicability of the results.

## CONCLUSIONS

This is the first study which pays attention to how Chinese patients with PCD perceive the impact of depression and anxiety on CVD and their preferences for treatment. We found that different illness comprehension levels are associated with variations in treatment willingness, considerations, health care preferences and medication choices. The education of cardiologists, as well as community physicians and CVD patients, should be strengthened to increase the recognition and treatment rate of PCD.

## Additional material


Online Supplementary Document
