# Supplementary material for: Analysis of the characteristics and illness comprehension bias among Chinese patients with psycho-cardiovascular disease: a multi-centre cross-sectional survey
Source: J Glob Health. 2025 Jan 31;15:04019. doi: 10.7189/jogh.15.04019 (PMC11781808; doi:10.7189/jogh.15.04019)
Supplement: Online Supplementary Document [file jogh-15-04019-s001.pdf]

JOURNAL OF GLOBAL HEALTH – SUPPLEMENT

Supplement to: Shi Z, Xia K, Li J, Lu J, Lu H, Li Y, Zhang J, Chen Q, Liu J, Ding R. Analysis of characteristics and illness comprehension bias among Chinese patients with psycho-cardiovascular disease: a multi-centre cross-sectional survey. J Glob Health. 2025;15:04017.

Table S1. Basic information and medical history of patients with PCD*

Items	Overall (n = 834)
Gender	
Male	429 (51.40)
Female	405 (48.60)
Height in cm	
$\bar{x}$ (SD)	167.00 (8.81)
MD (IQR)	167.00 (144.00–195.00)
Missing	4 (0.50)
Weight in kg	
$\bar{x}$ (SD)	70.70 (12.80)
MD (IQR)	70.00 (45.00–119.00)
Missing	8 (1.00)
BMI in kg/m^2	
<24	307 (36.80)
24–28	397 (47.60)
≥28	121 (14.50)
Missing	9 (1.10)
Marital status	
Married	714 (85.60)
Single	115 (13.80)
Missing	5 (0.60)
Medical insurance	
Yes	688 (82.50)
No	116 (13.90)
Missing	30 (3.60)
Age in years	
30–40	47 (5.60)
41–60	425 (51.00)
≥61	359 (43.00)
Missing	3 (0.40)
Occupation	
Labourer	188 (22.50)
National Business unit personnel	264 (31.70)
Unemployed	379 (45.40)
Missing	3 (0.40)

Education level	
Primary school or below	81 (9.70)
Secondary school	417 (50.00)
University	314 (37.60)
Master's degree or above	15 (1.80)
Missing	7 (0.80)
Monthly household income in CNY	
<4000	189 (22.70)
4000–7999	435 (52.20)
8000–12 000	160 (19.20)
>12 000	45 (5.40)
Missing	5 (0.60)
Region	
North	479 (57.40)
South	355 (42.60)
Three-question symptom†	
$\bar{x}$ *(SD)*	2.16(0.84)
MD (IQR)	2.00 (0.00–3.00)
Cardiovascular-related medical history‡	
$\bar{x}$ *(SD)*	3.04 (1.29)
MD (IQR)	3.00 (1.00–8.00)
Duration of illness in years	
<1	74 (8.90)
1–3	432 (51.80)
4–10	243 (29.10)
>10	85 (10.20)
Psychological consultation	
Yes	126 (15.10)
No	708 (84.90)
Psychological Therapy	
Yes	43 (5.20)
No	791 (94.80)
Impact of psychological factors on CVD	
Noticeable	211 (25.30)
Certain	463 (55.50)
Minor	142 (17.00)
No impact	10 (1.20)
Missing	8 (1.00)
Current efficacy evaluation	
Very satisfied	150 (18.00)
Satisfied	338 (40.50)
General	312 (37.40)
Not satisfied	30 (3.60)
Very dissatisfied	4 (0.50)
Comprehension degree of PCD	

Very familiar	56 (6.70)
Familiar with, but never in touch with	385 (46.20)
Not familiar with, but interested	351 (42.10)
Not familiar with and not interested	39 (4.70)
Missing	3 (0.40)
GAD-7 scores	
$\bar{x}$ (SD)	8.29 (2.15)
MD (IQR)	8.00 (5.00–16.00)
Degree of anxiety	
Mild	609 (73.00)
Moderate	218 (26.10)
Severe	7 (0.80)
PHQ-9 scores	
$\bar{x}$ (SD)	9.81 (2.65)
MD (IQR)	10.00 (5.00–20.00)
Degree of depression	
Mild	401 (48.10)
Moderate	394 (47.20)
Moderate to severe	38 (4.60)
Severe	1 (0.10)

BMI – body mass index, CVD – cardiovascular disease, GAD-7 – seven-item General Anxiety Disorder scale, IQR – interquartile range, MD – median, PCD – psycho-cardiovascular disease, PHQ-9 – nine-item Patient Health Questionnaire, SD – standard deviation, $\bar{x}$ – mean

*Presented as n(%) unless specified otherwise.

†The ‘three-question symptom’ is a method used by Chinese cardiologists to initially screen for possible CVD and mental health problems during or before diagnosis. The ‘three questions’ are as follows: 1) Is there poor sleep that has significantly affected the mental state during the day or requires medication? 2) Is there a feeling of restlessness and loss of interest in previously interesting things? 3) Is there any obvious physical discomfort, but multiple examinations have not found any reason to explain organic cardiovascular disease? If two out of three questions are answered with ‘yes,’ the likelihood of mental disorders is about 80% [20].

‡The cardiovascular-related medical history includes a history of drug treatment for CHD, heart valve replacement, stent/bypass surgery, heart failure, arrhythmia, peripheral artery

disease, cerebrovascular disease, hypertension, diabetes, dyslipidaemia, and other related conditions.

Table S2. Univariate and multivariate logistic regression analysis of influencing factors for anxiety in patients with PCD

	Anxiety			
**Characteristics**	**Univariate analysis, OR (95% CI)**	***P*-value**	**Multivariate analysis, OR (95% CI)***	***P*-value**
BMI in kg/m2†				
<24	Ref.			
24–28	1.09 (0.78–1.55)	0.609		
≥28	1.94 (1.24–3.03)	0.004		
Age in years				
30–40	Ref.			
41–60	1.76 (0.84–4.16)	0.160		
≥61	1.99 (0.95–4.73)	0.088		
Occupation†				
Labourer	Ref.			
National business unit personnel	0.58 (0.38–0.88)	0.010		
Unemployed	0.82 (0.56–1.20)	0.303		
Education level†				
Primary school or below	Ref.			
Secondary school	1.33 (0.79–2.29)	0.297		
University	0.66 (0.38–1.18)	0.154		
Master's degree or above	0.19 (0.01–1.03)	0.119		
Monthly household income in CNY				
<4000	Ref.		Ref.	
4000–7999	0.96 (0.67–1.41)	0.847	1.26 (0.83–1.94)	0.281
8000–12 000	0.69 (0.42–1.12)	0.139	0.90 (0.51–1.55)	0.698
>12 000	0.36 (0.13–0.85)	0.030	0.44 (0.15–1.13)	0.106
Duration of illness in years†				
<1	Ref.		Ref.	
1–3	0.37 (0.23–0.62)	<0.001	0.49 (0.27–0.88)	0.016
4–10	0.49 (0.29–0.84)	0.009	0.51 (0.28–0.94)	0.032
>10	0.62 (0.33–1.17)	0.141	0.62 (0.30–1.28)	0.198
Gender				
Male	Ref.			
Female	0.98 (0.73–1.34)	0.921		
Marital status				
Married	Ref.			
Single	1.32 (0.86–2.01)	0.196		
Medical insurance†				
No	Ref.			
Yes	3.45 (1.96–6.56)	<0.001		
Psychological consultation†				

No	Ref.		Ref.	
Yes	0.17 (0.08–0.33)	<0.001	0.40 (0.17–0.82)	0.018
Psychological therapy†				
No	Ref.			
Yes	0.42 (0.16–0.94)	0.053		
Region†				
North	Ref.		Ref.	
South	0.07 (0.04–0.11)	<0.001	0.08 (0.04–0.13)	<0.001
Three-question symptom†	0.82 (0.68–0.98)	0.030		
Cardiovascular-related medical history	0.83 (0.74–0.94)	0.004		
Comprehension degree of PCD				
Low	Ref.			
High	0.91 (0.67–1.24)	0.549		
Impact of psychological factors on CVD†				
No impact	Ref.			
Minor	0.90 (0.21–6.17)	0.894		
Certain	1.42 (0.35–9.47)	0.662		
Noticeable	2.12 (0.52–14.31)	0.348		

BMI – body mass index, CI – confidence interval, CVD – cardiovascular disease, OR – odds ratio, PCD – psycho-cardiovascular disease, ref – reference

*The final covariates were included through a stepwise regression approach.

†A statistically significant difference in the level of anxiety associated with this characteristic.

Table S3. Univariate and multivariate logistic regression analysis of influencing factors for depression in patients with PCD

	Depression			
**Characteristics**	**Univariate analysis, OR (95% CI)**	***P*-value**	**Multivariate analysis, OR (95% CI)***	***P*-value**
BMI in kg/m2				
<24	Ref.			
24–28	1.09 (0.81–1.46)	0.566		
≥28	1.29 (0.86–1.95)	0.220		
Age in years†				
30–40	Ref.			
41–60	0.54 (0.29–0.98)	0.044		
≥61	0.38 (0.20–0.69)	0.002		
Occupation				
Labourer	Ref.			
National business unit personnel	0.97 (0.68–1.40)	0.879		

Unemployed	0.82 (0.58–1.16)	0.262		
Education level†				
Primary school or below	Ref.			
Secondary school	1.51 (0.95–2.43)	0.084		
University	1.11 (0.69–1.80)	0.673		
Master's degree or above	0.45 (0.12–1.45)	0.205		
Monthly household income in CNY†				
<4000	Ref.			
4000–7999	1.10 (0.79–1.53)	0.587		
8000–12 000	0.46 (0.30–0.70)	<0.001		
>12 000	0.14 (0.06–0.32)	<0.001		
Duration of illness in years†				
<1	Ref.		Ref.	
1–3	0.94 0.58–1.52)	0.796	0.70 (0.41–1.17)	0.173
4–10	0.82 (0.49–1.36)	0.441	0.79 (0.46–1.37)	0.397
>10	0.49 (0.26–0.90)	0.022	0.29 (0.15–0.58)	<0.001
Gender				
Male	Ref.			
Female	0.88 (0.67–1.15)	0.348		
Marital status				
Married	Ref.			
Single	1.12 (0.76–1.65)	0.571		
Medical insurance†				
No	Ref.		Ref.	
Yes	0.45 (0.30–0.66)	<0.001	0.66 (0.41–1.06)	0.088
Psychological consultation†				
No	Ref.		Ref.	
Yes	2.23 (1.54–3.25)	<0.001	1.55 (0.99–2.45)	0.057
Psychological therapy				
No	Ref.			
Yes	0.61 (0.32–1.12)	0.118		
Region†				
North	Ref.		Ref.	
South	0.73 (0.56–0.96)	0.024	0.38 (0.27–0.52)	<0.001
Three-question symptom†	1.44 (1.23–1.71)	<0.001		
Cardiovascular-related medical history†	1.57 (1.41–1.75)	<0.001	1.62 (1.41–1.86)	<0.001
Comprehension degree of PCD†				
Low	Ref.			
High	1.72 (1.32–2.26)	<0.001		
Impact of psychological factors on CVD†				
No impact	Ref.			
Minor	0.82 (0.23–3.28)	0.760		

Certain	1.83 (0.54–7.15)	0.345
Noticeable	2.38 (0.69–9.42)	0.182

BMI – body mass index, CI – confidence interval, CVD – cardiovascular disease, OR – odds ratio, PCD – psycho-cardiovascular disease, ref – reference

*The final covariates were included through a stepwise regression approach.

† A statistically significant difference in the level of depression associated with this characteristic.
